# Characterization of environmental drivers influencing the abundance of *Anopheles maculipennis* complex in Northern Italy

**DOI:** 10.1186/s13071-024-06208-6

**Published:** 2024-03-06

**Authors:** Gianni Gilioli, Francesco Defilippo, Anna Simonetto, Alessandro Heinzl, Manlio Migliorati, Mattia Calzolari, Sabrina Canziani, Davide Lelli, Antonio Lavazza

**Affiliations:** 1 Department of Civil Engineering Architecture Land and Environment and Mathematics, University of Brescia, Brescia, Italy; 2 Institute Zooprofilattico Sperimentale Della Lombardia E Dell’Emilia Romagna, Brescia, Italy; 3 Institute Zooprofilattico Sperimentale Della Lombardia E Dell’Emilia Romagna, Reggio Emilia, Italy

**Keywords:** Malaria, *Anopheles*, Land use, Meteorological variables

## Abstract

**Background:**

In Italy, malaria was endemic until the 1970s, when it was declared eradicated by WHO. Nowadays, with the persistence of competent mosquito populations, the effect of climate change, and increased possibility of importing malaria parasites from endemic counties due to growing migration, a malaria resurgence in Italy has become more likely. Hence, enhancing the understanding of the current distribution of the *Anopheles maculipennis* complex and the factors that influence the presence of this malaria vector is crucial, especially in Northern Italy, characterised by a high density of both human population and livestock.

**Methods:**

To assess the presence and abundance of malaria vectors, a 4-year field survey in the plain areas of Lombardy and Emilia-Romagna region in Italy was conducted. Every sampling point was characterised in space by the land use in a 500-m radius and in time considering meteorological data collected in the short and long time periods before sampling. We combined the results of a linear regression model with a random forest analysis to understand the relative importance of the investigated niche dimensions in determining *Anopheles* mosquito presence and abundance.

**Results:**

The estimated normalised variable importance indicates that rice fields were the most important land use class explaining the presence of *Anopheles*, followed by transitional woodlands and shrubland. Farm buildings were the third variable in terms of importance, likely because of the presence of animal shelters, followed by urbanised land. The two most important meteorological variables influencing the abundance of *Anopheles* in our study area were mean temperature in the 24 h before the sampling date and the sum of degree-days with temperature between 18 °C and 30 °C in the 14 days before the sampling date.

**Conclusions:**

The results obtained in this study could be helpful in predicting the risk of autochthonous malaria transmission, based on local information on land cover classes that might facilitate the presence of malaria vectors and presence of short- and medium-term meteorological conditions favourable to mosquito development and activity. The results can support the design of vector control measures through environmental management.

**Graphical Abstract:**

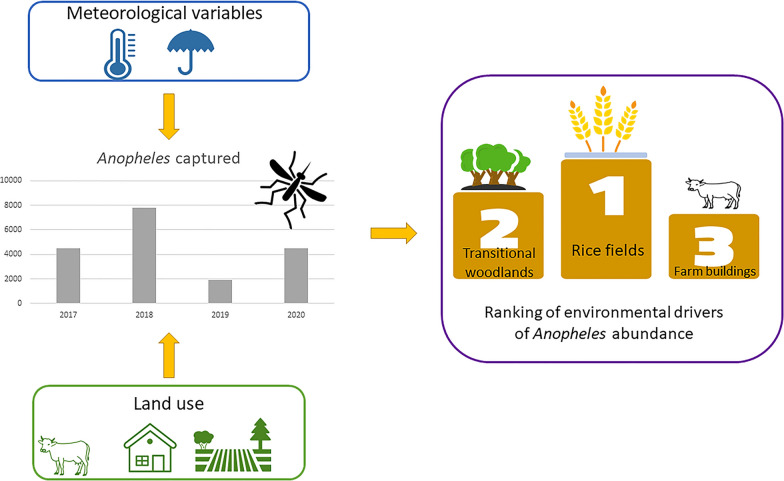

**Supplementary Information:**

The online version contains supplementary material available at 10.1186/s13071-024-06208-6.

## Background

Climate change affects the transmission dynamics and geographic distribution of vector-borne diseases. Insect vector species are ectotherms, and the temperature increase affects their abundance, survival, and feeding activity as well as the pathogen development within the vector itself, reducing the duration of the incubation period [1]. The rapid expansion of global travel and trade increases the probability of travel by infected persons, who can be responsible for local outbreaks of severe diseases in non-endemic areas; the outbreaks of chikungunya in Italy in 2007 and 2017 [2, 3], dengue in Croatia [4], and West Nile virus in Romania in 1996 [5] are examples of the spreading capacity of these vector-borne diseases.

The increase of travellers and migratory flows to and from endemic areas make malaria the predominant imported infectious disease in non-endemic countries [6]. This is the case, for example, in Europe [7], which has fought malaria for centuries. However, malaria is still endemic in the coastal and inland plains of Asian areas of Turkey [8], and cases of local transmission in Greece are still reported [9]. Italy was one of the last countries where malaria was eradicated through a massive campaign treating infected human hosts and using effective insecticides. The combination of these efforts led to WHO's 1970 declaration that Italy is a malaria-free country [7]. Despite this, mosquito species of the genus *Anopheles* (which includes the main vectors of *Plasmodium* species responsible for human malaria) are still present in many suitable areas in Italy [8, 10–13]. In northern Italy, the Anophelinae of the Maculipennis complex is a group comprising both primary malaria vectors and species of low/negligible epidemiological importance. Recent studies have shown that the most abundant species of this complex in Pianura Padana, the main lowland of Italy, are *Anopheles messeae*, followed by *An. maculipennis* s.s., *An. atroparvus*, and *An. melanoon* [14].

Italian health institutions constantly monitor the possibility that an imported pathogen has activated a chain of local infections. The last episode of local transmission in Italy dates back to 1997 [15], but a local cluster of nosocomial malaria was reported in 2017 [6]. Malaria occurrence and epidemiological patterns are greatly influenced by environmental variables and land use patterns [16]. Multiple factors, including precipitation, temperature, altitude, and human population density, can act as risk components influencing the transmission process of malaria through sources of infection, transmission routes, and susceptible individuals [17]. Currently, only a few studies have been conducted to identify the broader environmental conditions influencing *An. maculipennis* complex vector presence and abundance in Italy as a risk factor for malaria transmission in this county [18]. For example, to evaluate the risk of malaria transmission in central Italy, Romi et al. [19] combined a multifactorial approach with climatic parameters. However, as pointed out by Boccolini et al. [6], there is a serious knowledge gap in an accurate and updated distribution map of Italian anopheline mosquitoes; this is especially relevant because of the recent climate and environmental changes, extending their geographical range beyond previous that of records.

This study aims to investigate the environmental factors (land use and meteorological variables) that mainly influence local presence and abundance of *An. maculipennis* complex in the Lombardy and Emilia-Romagna regions (Northern Italy) using data from the West Nile disease surveillance plan from 2017 to 2020. The entomological surveillance systems in the two Italian regions were based on the same attraction trap model and used the same sampling periodicity in summer. The Lombardy and Emilia-Romagna regions were characterised in the past by a high population abundance of *Anopheles* mosquitoes, and the environmental conditions are still locally suitable for the malaria vectors [14]. For example, Lombardy has 60% of Italy's rice fields, while Emilia-Romagna has a wide wetland area associated with the Po River Delta. Moreover, 24% of the Italian population (14.4 million people in an area of 46,354 km^2^) lives in these two regions. The results of our study can help to understand the population distribution of competent malaria vectors in northern Italy to plan better prevention and control activities for managing the risk of local transmission of malaria imported from endemic areas.

## Methods

### Study area

Samples of mosquitoes belonging to the *Anopheles* genus were retrieved from an entomological monitoring programme for the epidemiological surveillance of West Nile disease (WND) in Lombardy and Emilia-Romagna regions (Northern Italy). The study area covers approximately 28,000 km^2^ and involves a large part of the Po Valley, the most important Italian floodplain, characterised by intensive agriculture and animal husbandry. Annual crops are the most important component in the farming systems; vineyards, orchards, and horticulture are locally abundant. The two monitored regions are densely populated, with many cities, villages, and industrial areas. The eastern part of the Emilia-Romagna is located on the Adriatic Sea and is characterised by the presence of large natural wetlands (Po River Delta and Valli di Comacchio). The Po Valley is delimited by two mountain chains, the Apennines in the south and the Alps in the north, representing unsuitable areas for mosquitoes due to the altitude, although the valleys can act as important places for the mosquitoes to spread. The climate of the northern and southwestern part of the study area (Lombardy) is classified as humid continental; in the southeastern part (Emilia-Romagna), the climate is typically sub-continental, gradually becoming sub-Mediterranean towards the coastal part of the region.

### Sampling design and mosquito collections

**Fig. 1:**
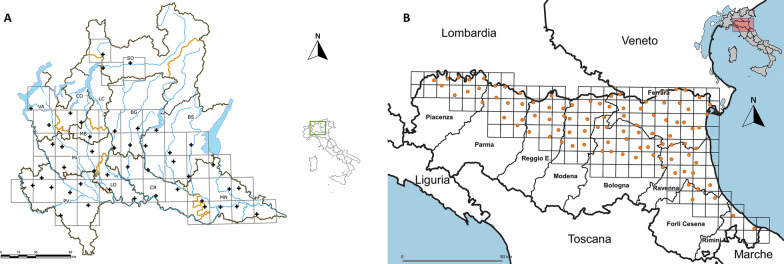
Distribution of the sampling sites for the West Nile virus surveillance programme in **A** Lombardy and **B** Emilia-Romagna. Sampling points are represented by black crosses in Lombardy (above) and by orange dots in Emilia-Romagna (below)

Mosquitoes were collected in a 4-year period from 2017 to 2020. The sampling area covers the whole plain area of the two regions, with some exceptions as found by traps placed in the province of Sondrio (Fig. 1a). The sampling area was subdivided in two regular grids: in 44 cells of 400 km^2^ each in Lombardy (Fig. 1a) and 95 cells of 110 km^2^ each in Emilia-Romagna (Fig. 1b). Traps were placed in sites considered of local risk (i.e. abundance of potential breeding sites) or in which previous West Nile virus circulation had been reported. Sampling sites were selected mainly in rural areas in Lombardy and semi-natural, rural, or peri-urban areas in Emilia-Romagna. Mosquitoes were trapped during the night from 17:00 until 9:00 every 2 weeks from June to September, using CDC attractive traps baited with dry ice as a source of carbon dioxide at fixed stations. In the trap catches *Anopheles* specimens were separated from other mosquitoes according to morphological taxonomic keys [10, 20].

The meteorological variables considered in the analysis were air temperature, dew point, and rainfall. Meteorological variables can impact mosquitoes’ life history parameters (e.g. development, survival, and fertility rates) and behavioural parameters (e.g. flight pattern, biting rate) influencing population abundance and activity. Therefore, we used two different time periods for the meteorological variables. To account for the influence of meteorological variables on life history parameters, a period of 14 days before the sampling date was considered (hereafter, ‘long term’). The meteorological variables in the 24-h period before the sampling (hereafter, ‘short term’) were considered to influence the mosquitoes' behaviour. Two derived meteorological variables were considered for their influence on mosquito life history. The first is defined as T_w_ and accounts for the suitable heat accumulation for the development and is calculated as the cumulative degree-days in the 14 days before the sampling event setting with the lower thermal threshold at 18 ℃ and the upper thermal threshold at 30 °C. The second variable is defined as T_h_ and accounts for the thermal stress due to high temperature; it is calculated in terms of the cumulated degree-days equal to or above a threshold of 32 ℃ [21]. The meteorological and derived variables used in the study are detailed in Table 1.

**Table 1:** Variables considered in this study to account for the influence of meteorology of the sampling sites on life history parameters (calculated in a 14-day period before the sampling date, ‘long-term’ period) and on the mosquitoes’ behaviour (calculated in the 24 h before the sampling, ‘short-term’ period)

Period	Variable	Code	Units
Short term	Mean of the hourly temperature for 24 h before the sampling date	T_s_	°C
	Cumulated rain for 24 h before the sampling date	R_s_	mm
	Mean of hourly air relative humidity for 24 h before the sampling date	H_s_	%
Long term	Mean of daily temperature in the 14 days before the sampling date	T_l_	°C
	Cumulated rain in the 14 days before the sampling date	R_l_	mm
	Mean hourly air relative humidity in the 14 days before the sampling date	H_l_	%
	Sum of degree-days with temperature between 18 °C and 30 °C in the 14 days before the sampling date	T_w_	Degree-days
	Sum of degree-days > 32 °C in the 14 days before the sampling date	T_h_	Degree-days

Meteorological variables were extracted from the ERA5-Land database, which provides a reanalysed dataset at a high spatial (9 km grid) and temporal (hourly) resolution [22]. To obtain meteorological data at the specific sampling point and time, the ERA5-Land gridded data were interpolated on the sampling points using bilinear interpolation through Climate Data Operators (CDO) [23].

### Land use

For each sampling site, we characterised the land use in a circular area with a 500-m radius [24], according to Taconet [25]. Data on land use were retrieved from two geodatabases, the DUSAF 6 (‘*Destinazione d’Uso dei Suoli Agricoli e Forestali*’) for the Lombardy region [26] and the ‘*Coperture vettoriali uso del suolo di dettaglio*’ for the Emilia-Romagna region [27]. Both geodatabases have a hierarchical structure with five nested classification levels. The first three levels are mainly based on the Corine Land Cover classification [28]; the fourth and fifth levels, those with greater detail, have been differently specified by the two regional systems. To proceed with the analysis, we created a common classification of land use types by combining the two regional classifications. We obtained 25 types of land use, detailed in the Additional materials (see Additional file 1: Table S1) (Table 2).

**Table 2:** Abundance of *Anopheles maculipennis* complex in Northern Italy

Area	Number of sites with at least one *Anopheles*	Number of samples with *Anopheles*	*Anopheles* abundance	
			Mean (SD)	[min; max values]
Lombardy	53	343	47.63 (148.41)	[0; 1639]
Emilia-Romagna	76	517	4.55 (8.57)	[0; 100]
Whole area	129	859	21.72 (96.27)	[0; 1639]

For each region, the number of samplings with the presence of *Anopheles* in the 4-year sampling period, the mean value of specimens collected per sampling, the standard deviation (SD), and the minimum and maximum values are reported

### Statistical analysis

To investigate relationships between the abundance of *Anopheles maculipennis* complex and environmental (i.e. meteorological and land use) variables, we combined the simplicity and easiness of interpretation of linear regression (LR) with the high accuracy of the random forest analysis (RF) [29]. In both models, to account for the skewness of data, we considered the logarithm base 10 of the abundance of *An. maculipennis* complex in the site per sampling data as the dependent variable. Before fitting the regression models, we preliminary investigated the linear correlations between the environmental regressors and excluded variables presenting Pearson correlation coefficient S > 0.7. In the LR analysis, we applied the stepwise approach to select the environmental regressors according to the Akaike information criterion (AIC). We studied multicollinearity among regressors using variance inflation factor (VIF), excluding variables with VIF value > 5 to eradicate strong dependence among regressors.

The variable importance measures, i.e. the relative influence of a regressor in predicting the response variable [30], of LR and RF are used to merge the results of the two models. First, we normalised the variable importance estimated within each model. The highest value was set equal to 100, and other values were proportionally rescaled, with variable importance for not used regressors set equal to 0. Then, we considered the average between LR and RF values for each regressor. All the statistical analyses were performed in the R environment [31], using functions lm for LR and randomForest (package randomForest) [32] for RF.

## Results

In the 4-year of survey period, a total of 1,365,958 mosquitoes of different species were collected (264,453 in Lombardy and 1,101,505 in Emilia-Romagna), of which 18,637 females belonged to the *An. maculipennis* complex (16,289 in Lombardy and 2348 in Emilia-Romagna). Only samplings with presence of at least a single *An. maculipennis* complex mosquito (positive samplings) were included in the analysis. The original dataset is related to the West Nile disease surveillance plan; unfortunately, in this context, not all the observations with a 0 abundance of *Anopheles* were reported in the dataset. Consequently, we had to remove the few available observations for consistency. In total, for 3263 total samplings, there were 859 positive samples (26.3%).

The number of *Anopheles* caught was much higher in Lombardy than in Emilia-Romagna; this could be explained by the large representation of farms in the sampling locations in Lombardy where the typical farm combines crop area and animal husbandry, which are particularly attractive for *An. maculipennis* complex.

The descriptive statistics of the land use surrounding the sampling sites and the meteorological variables are reported in Tables 3 and 4. Based on the analysis of the correlation matrix, we (i) removed four meteorological variables (H_s_, T_l_, H_l_, T_h_), (ii) merged ‘broad-leaf forest’, ‘coniferous forest’, and ‘mixed forests are joined’ in the new land use category ‘forest’, and (iii) merged ‘inland marshes’ and ‘salt marshes’ in the new land use category ‘wetlands’. Therefore, we finally obtained 25 regressors (4 meteorological variables and 21 land use categories) for the LRM analysis and the RF.

**Table 3:** Descriptive statistics of the top ten land use categories in terms of average buffer coverage (% of the circular area around the sampling point with a radius of 500 m)

	% of buffer area covered by the land use category	
	Mean (SD)	Median [min; max]
Annual crops	52.46 (25.50)	5.92 [0.69; 97]
Urban area	10.38 (11.86)	5.27 [0; 56.06]
Permanent crops	5.95 (9.86)	1.43 [0; 54.12]
Water courses	4.2 (8.61)	0.66 [0; 58.87]
Industrial or commercial units	3.72 (8.15)	0.52 [0; 73.01]
Broad-leaf forest	2.92 (8.11)	0.00 [0; 63.21]
Road and rail networks and associated land	2.87 (4.94)	0.95 [0; 26.24]
Inland marshes	2.58 (8.89)	0.00 [0; 57.04]
Pastures	2.52 (6.07)	0.00 [0; 44.04]
Green urban areas	1.94 (4.08)	0.01 [0; 24.54]

*SD* standard deviation

**Table 4:** Descriptive statistics of meteorological and derived variables

Variables and [units]	Mean (SD)	Median [min; max]
T_s_ [°C]	24.18 (1.16)	25.93 [12.98; 33.20]
R_s_ [mm]	0.03 (0.02)	0.01 [0.00; 1.00]
H_s_ [%]	65.28 (4.54)	66.34 [38.77; 90.59]
T_l_ [°C]	23.90 (1.25)	24.43 [12.98; 28.92]
R_l_ [mm]	0.47 (0.12)	0.26 [0.00; 3.56]
H_l_ [%]	65.46 (4.54)	63.60 [45.57; 84.68]
T_h_ [degree-days]	1.15 (0.02)	0.01 [0.00; 17.19]
T_w_ [degree-days]	66.83 (1.56)	79.17 [2.47; 165.87]

*SD* standard deviation

The linear regression model with stepwise procedure selected 13 regressors (Table 5) as those statistically significant to explain the variability of the log abundance of *An. maculipennis* complex females in the samplings (the adjusted R^2^ is 0.2096).

**Table 5:** Results of the linear regression model for the log abundance of *Anopheles*. VI norm.: normalised variable importance measure

Regressor	Regression coefficient estimates	*t*-statistics	*P*-value	VI norm
Rice fields	0.016	6.312	< 0.001	100
Farm buildings	0.034	5.316	< 0.001	84
Transitional woodland shrub environment	0.017	4.732	< 0.001	75
Road and rail networks and associated land	− 0.016	− 4.295	< 0.001	68
T_w_	0.004	3.998	< 0.001	63
Urban area	0.007	3.961	< 0.001	63
Water courses	− 0.008	− 3.67	< 0.001	58
T_s_	0.019	2.714	0.007	43
Port areas	1.100	2.244	0.025	36
Pastures	− 0.009	− 2.278	0.023	36
R_s_	− 0.570	− 2.192	0.029	35
Graveyard	0.078	1.795	0.073	28
Permanent crops	0.003	1.735	0.083	27

Four regressors had a negative estimated coefficient (‘road and rail networks and associated land’, water courses, pastures, and R_s_), indicating a negative relationship between the regressor and average expected abundance of *An. maculipennis* complex. The *p*-values are all < 0.1, with eight *p*-values < 0.01 and three *p*-values < 0.05, so we can consider all estimated coefficients to be statistically different from 0. According to the normalised variable importance (VI norm), the most important regressor is ‘rice fields’.

The random forest model showed a better fitting to the data than the linear regression model, with an explained variance equal to 42.89%. The normalised variable importance for the random forest model is reported in Table 6. ‘Rice fields’ is the most important variable in explaining the variability of the target variable (in accordance with the linear model), followed by ‘transitional woodland shrub environment’ and two meteorological regressors, T_s_ and ‘T_w_. Only three regressors (‘beaches, dunes, sand plains’, ‘airports’, and ‘port areas’) have a normalised variable importance equal to 0, indicating that they are not selected by the model to explain the variability in the abundance of *An. maculipennis* complex mosquitoes.

**Table 6:** Normalised variable importance (VI norm) values of the explanatory variables used in the random forest

Regressor	VI norm	Regressor	VI norm
Rice fields	100	Sport and leisure green areas	7
Transitional woodland shrub environment	55	R_s_	6
T_s_	40	R_l_	6
T_w_	24	Green urban areas	5
Urban area	17	Industrial or commercial units	4
Annual crops	16	Mine, dump, and construction sites	3
Permanent crops	16	Graveyard	3
Farm buildings	14	Wetlands	3
Water courses	13	Water bodies	1
Pastures	11	Port areas	0
Forest	9	Airports	0
Road and rail networks and associated land	8	Beaches, dunes, sand plains	0

**Fig. 2:**
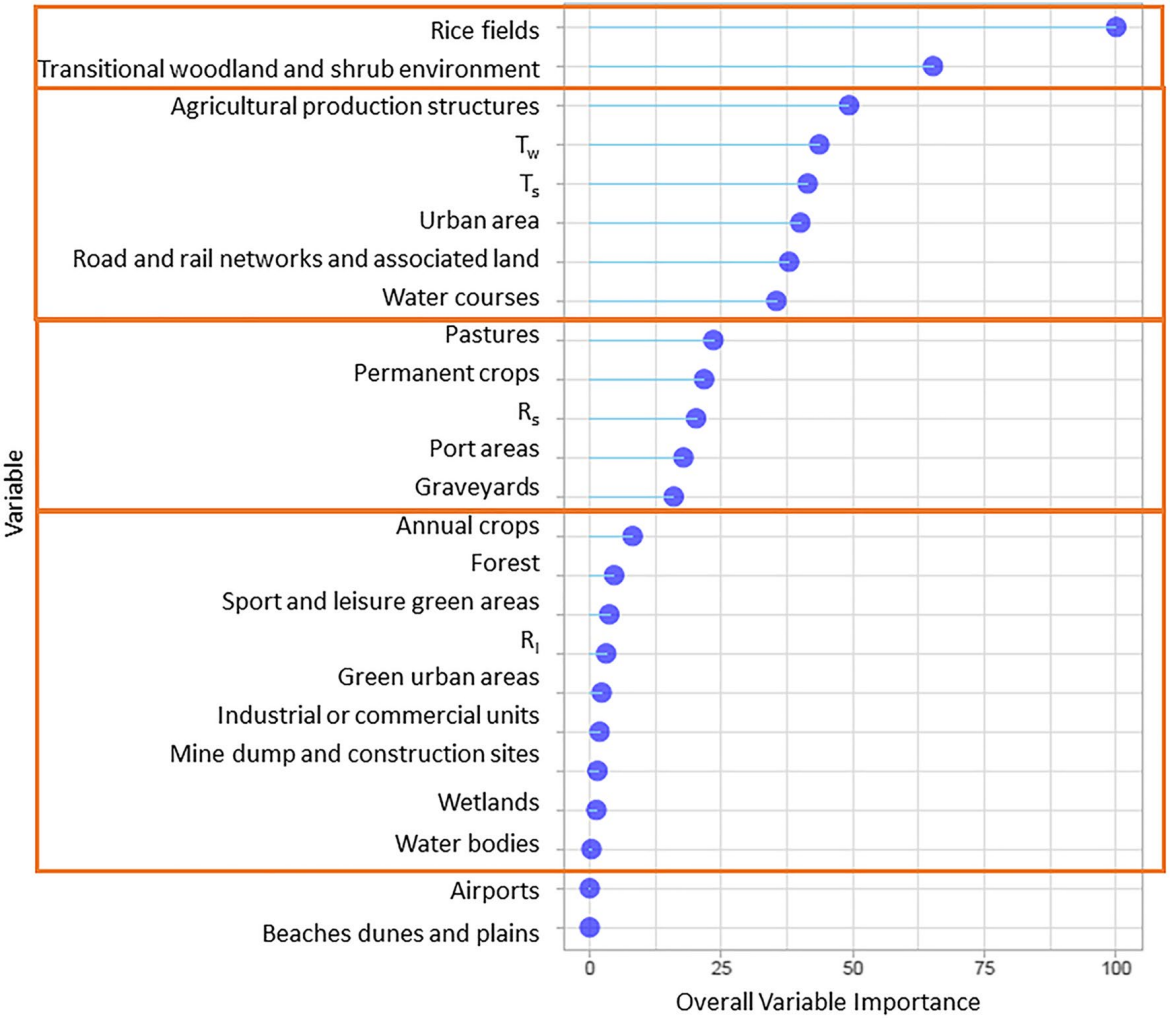
Overall variable importance estimates for *Anopheles* abundance in the study area combining results of the linear regression and random forest for the set of variables investigated. The orange rectangles cluster variables with similar importance

To integrate the results of the linear regression and the non-linear regression (random forest) model, the overall variable importance is calculated (Fig. 2). The overall variable importance is set equal to 0 for regressors not used by both models (‘beaches, dunes, sand plains’ and ‘airports’) and equal to 100 for the most important regressor selected by both models (‘rice fields’). ‘Transitional woodland shrub environment’ was the second most important variable. Then, we identified three groups of regressors: the medium importance (6 variables with overall variable importance between 35 and 50), low importance (5 variables with overall variable importance between 12 and 25), and very low importance (9 variables with overall variable importance between 1 and 10). Results are reported in Fig. 2.

The two single-model rankings of variable importance showed good accordance, except for ‘farm buildings’, classified as the eighth most influential regressor in the random forest analysis (normalised VI = 14) and second in the linear regression model (normalised V = 84), and for ‘roads and rail networks and associated land’, which had a high normalised importance in the linear regression analysis and a low one in the random forest analysis (68 and 8, respectively).

## Discussion

Our analysis identified a clear ranking in the importance of the variable investigated and allowed grouping them in four classes: high, medium, low, and very low importance. There was also a fifth class grouping the variables with no importance in our study area.

In the high importance class, we reported the presence of rice fields in the surroundings of the sampling site as the most critical factor positively influencing the abundance of *An. maculipennis* complex collected. This result is consistent with several studies considering rice fields as the main breeding site for *Anopheles* in Italy [33–35], and it supports previous findings demonstrating that the abundance of the Maculipennis complex is particularly high in sites characterised by extensive breeding grounds, such as rice fields and wetlands [14]. Both regression models highlighted the great importance of transitional woodland and shrub habitats in positively influencing the presence of *Anopheles*. This result is consistent with similar findings, as the one reported in Mali, where removing a flowered invasive shrub resulted in a 69.4% drop in mosquito population density [36]. Multiple factors could be involved in explaining our observations. In the study area, transitional woodlands and shrubs often represent the interface between the rural and urban environments. These habitats can guarantee good resting sites to adult mosquitoes and are frequently and rapidly colonised by plants rich in sugars, such as nectar and gland exudates (e.g. *Ailanthus altissima* and *Robinia pseudoacacia*), which can serve as a trophic source for the *Anopheles* mosquito.

In the medium importance class, we observed a positive relation between the abundance of *Anopheles* and the presence of ‘farm buildings’, in line with the well-known attractiveness of bovine herds for malaria vectors. In Ethiopia [37] and Malawi [38], it has been observed that proximity of cattle increases the abundance of *Anopheles*. In Europe, the importance of bovines was confirmed by a survey in a former endemic area in Romania, which found that 85.5% of captured mosquitoes fed on cattle [39]. Similar results (82.4% cattle, 5.1% human) have been obtained in a study performed in Corsica (France) [40]. Animal shelters are also used as resting sites. In previous studies, over a hundred females [40], up to 500 [41], were found in animal shelters. Although our results are in line with the literature, the importance attributed to ‘farm buildings’ in our study could be partially overestimated by the location of sampling points in the Lombardy region that were close to animal shelters.

In our study, thermal sum in the optimal range for development T_w_ (the sum of degree-days with temperature between 18 ℃ and 30 ℃ in the 14-day period before the sampling date) shows high variable importance. This is in accordance with literature where the importance of the thermal sum in the optimal range temperatures is confirmed for the development of eggs [42] and larval stages [43]. The mean temperature in the 24 h before the sampling, T_s_, is also a positive factor in our study since higher temperatures cause an increase in mosquito activity. The cumulative rain in the 24 h before the sampling (R_s_) was a negative factor affecting the probability of trapping mosquitoes. Although mosquitoes can fly during rain [44], some studies show that as rainfall increases the number of mosquitoes sampled decreases [45].

The presence of urban areas in the surroundings of the sampling points increases the probability of catching *An. maculipennis* complex. This is consistent with other studies in which a high abundance of *Anopheles* has been found even in cities, as in a survey in The Netherlands where *An. maculipennis* occurred in similar proportions in urban and agricultural areas [46].

The study identifies rails and railroads as disturbing elements that reduce the expected abundance of *Anopheles*. In other studies, they have been found to be a positive factor since roads usually have drainage areas along their margins that become transient water bodies during and after rain. The possible use of these areas as breeding sites was investigated in Ecuador, and they hosted species of the *Anopheles* genus such as *An. punctimacula* and* An. albimanus* [47]. This positive effect was not recorded in our study area.

Rivers can be used as breeding nesting sites by *Anopheles*, particularly in areas with slow water flow and the presence of riparian vegetation [43], as reported for example in surveys in Ecuador [47] and in Italy by Romi et al. [33], which found different species of *Anopheles* in riverine areas. These favourable riparian habitats are present in the study area; however, rivers are characterised by a negative coefficient in the regression analysis. A possible explanation for this result is the level of the pollutants characterising many rivers in the study area, which show low to moderate concentrations of heavy metals and organic micropollutants and high contents of nutrients causing eutrophication, as reported in Provini and Binelli [48]. Although in the literature *Anopheles* seems to show an increasing tolerance for organic pollutants [49], the contamination level of the river in the study area can be higher than its tolerance threshold.

The low importance attributed to port areas, in our study area fluvial ports, and the positive regression coefficient, in contrast to the negative coefficient of riverine areas, can be attributed to the fact that port areas are only present in a small fraction of our study area (< 0.01%).

In the low importance class, we observed a positive relationship between permanent crops and *Anopheles* presence, in agreement with other studies [41]. An example is the research conducted in Ghana [50], which found many suitable larval habitats in farmlands and pastures with abundant ditches, furrows, and drinking troughs. Similar results were found in a survey in The Netherlands [46]. In our study, pastures were a negative coefficient, which can be attributed to the small spatial extension of this area since in our study area intensive animal husbandry is mainly practised in shelters.

Our results show a positive moderate variable importance for graveyards in which there are constant artificial water reservoirs such as flower pots [51]. A review found that cemeteries are perfect breeding grounds for artificial container-breeding mosquitoes [52], which is not the case for *Anopheles*, even though *An. maculipennis* s.s. is considered more adapted to artificial environments than other species in the group [46].

In the very low importance class, we observed a cluster of very different variables. ‘Green urban areas’ belong to this class, although a meta-analysis on the abundance of mosquitoes comparing urban environments and green urban areas showed no significant difference between the two [53].

The very low importance of wetlands, although considered one of the most suitable habitats for *Anopheles* mosquitoes, can be attributed to the small presence of wetlands in our study and to the salinity of coastal wetlands, which characterises most of the wetlands close to the sampling points and can be above the tolerance threshold for *An. maculipennis* [40].

Airports, beach dunes, and plains were land use classes of no importance in our study because of their high artificial or natural draining capacity.

## Conclusions

Serious knowledge gaps were mainly found in entomological risk assessment due to (i) lack of accurate and up-to-date distribution maps of Italian Anopheline mosquitoes, which have most likely changed their geographic range in recent decades because of environmental and climatic changes; (ii) limited knowledge on the susceptibility of current Anopheline populations to infection by different species and geographic populations of imported *Plasmodium *[6].

Our study provides data on the distribution and abundance of Anopheline species in two extensive regions of northern Italy, contributing to filling the knowledge gaps on malaria vectors in this area. The results we obtained allowed us to characterise environmental drivers influencing the presence and the abundance of the *An. maculipennis* complex. Quantitative information could help with the development of models predicting the risk of transmission of possible pathogens carried by this group of insects, including malaria, by knowing land cover types and the current status and past trends of meteorological variables. The results could also be applied to the design and implementation of vector control measures through environmental management methods to reduce the risk of human-mosquito contact.

### Supplementary Information

**Additional file 1: Table S1.** Classification of land use categories.

## Data Availability

The data supporting the findings of the study must be available within the article and/or its supplementary materials, or deposited in a publicly available database.
