# Supplementary material for: Characterization of environmental drivers influencing the abundance of Anopheles maculipennis complex in Northern Italy
Source: Parasit Vectors. 2024 Mar 6;17:109. doi: 10.1186/s13071-024-06208-6 (PMC10916043; doi:10.1186/s13071-024-06208-6)
Supplement: Supplementary file 1 — Additional file 1: Table S1. Classification of land use categories. [file 13071_2024_6208_MOESM1_ESM.docx]

# Additional materials

Table S1: Classification of land use categories. In the first coloumn the classification used in this study, obtained marging the DUSAF classification [25] (second column) and the ‘Coperture vettoriali uso del suolo di dettaglio’ [26]classification.

| **Merged classification** | **Dusaf classification** | **Emilia Romagna classification** |
| --- | --- | --- |
| Urban Area | 1111 Dense residential area  1112 Continuous residential area averagely dense  1121 Discontinuous residential area  1122 Sparse and nucleiform residential area  1123 Sparse residential area  11231 Farmhouses | 1111 Dense and compact residential area  1112 Sparse residential area  1121 Urban residential area  1122 Isolated residential structures |
| Industrial or commercial units | 1111 Dense residential area  1112 Continuous residential area averagely dense  1121 Discontinuous residential area  1122 Rare and nucleiform residential fabric  1123 Scattered residential fabric  11231 Farmhouses | 1111 Dense and compact residential area  1112 Sparse residential area  1121 Urban residential area  1122 Isolated residential structures |
| Farm buildings | 12112 Agricultural production units | 1212 Agro-zootecnical units  4213 Aquaculture |
| Graveyard | 12124 Graveyard | 1430 Graveyard |
| Road and rail networks and  associated land | 122 Road and rail networks and associated land  1221 Road network and auxiliary spaces  1222 Railways and auxiliary spaces | 1221 Highway  1222 Road network  1223 Green areas associated with viability  1224 Railways  1225 Goods sorting facilities  1226 Telecommunication plants  1227 Networks for energy distribution and production  1228 Photovoltaic systems  1229 Water distribution network |
| Port areas | 123 Port areas | 1231 Commercial port areas  1232 Recreational port areas  1233 Fishing port areas |
| Airports | 124 Airports and heliports | 1241 Commercial airports  1242 Airports for sport flying and heliports  1243 Military airports |
| Mine dump and construction sites | 131 Mineral extraction sites  132 Dump sites  133 Construction sites  134 Not used and not green degraded areas | 1311 Active extraction site  1312 Inactive extraction site  1321 Dumping site for quarries, mines  1322 Landfills  1323 Wreck deposit  1331 Construction sites  1332 reworked and artificial soils |
| Green urban areas | 1411 Parks and gardens  1412 Non cultivated green areas | 1411 Parks  1412 Mansions  1413 Urban non cultivated green areas |
| Sport and leisure green areas | 1421 Sport facilities  1422 Camping and tourist facilities  1423 Amusement parks  1424 Archeologic areas | 1421 Camping and tourist facilities  1422 Sport facilities  1423 Theme parks  1424 Golf courses  1425 Hippodromes  1426 Racetracks  1427 Archeologic areas  1428 bathing areas |
| Annual crops | 2111 Simple arable land in non-irrigated areas  2112 Wooded arable land  21131 Field scale horticultural crops  21132 Greenhouses.  21141 Flower field  21142 Greenhouse for flowers  2115 vegetable garden | 2121 Irrigated planted field  2122 Seedbed  2123 Vegetable field  2410 Temporary crops combined with permanent crops  2420 Complex crop and particle systems  2430 Agricultal lands associated with relevant natural areas |
| Rice fields | 213 Rice fields | 2130 Rice fields |
| Permanent crops | 221 Vineyards  222 Orchards and small fruits  223 Olive grove  2241 Poplar grove  2242 Other arboreal grove | 2210 Vineyards  2220 Orchards  2230 Olive grove  2241 Poplar grove  2242 Other arboreal grove |
| Pastures | 2311 Pastures without trees and shrubs  2312 Pastures with sparse trees and shrubs  2313 water meadow | 2310 Pastures |
| 311 Broad leaved forest | 3111 High and medium density broad leaved forest  31111 Managed high and medium density broad leaved forest  31112 High canopy managed high and medium density broad leaved forest  3112 Low density broad leaved forest  31121 Managed Low density broad leaved forest  31122 High canopy managed Low density broad leaved forest  3113 Riparian vegetation  3114 Chestnut grove | 3111 Beech forest  3112 Oak, *Carpinus* and chestnut forest  3113 Willow and poplar forest  3114 Oak forest  3115 Chestnut grove  3116 Relictous wood |
| Coniferous forest | 3121 High and medium density Coniferous forest  3122 Low density Coniferous forest | 3120 Coniferous forest |
| Mixed fores | 3131 High and ,medium density Mixed forest  31311 Managed high and medium density mixed forest  31312 High canopy managed high and medium density mixed forest  3132 low density density mixed forest  31321 Managed low density mixed forest  31322 - High canopy managed low density mixed forest  314 Recent reforestation | 3130 Mixed forest with coniferous and broad leaved trees |
| Transitional woodland shrub environment | 3211 High altitude pastures without trees and shrubs  3212 - High altitude pastures with trees and shrubs  3221 Shrubs  3222 Riparian vegetation  3223 Riparian vegetation of high embankments  3241 Shrubland with significative presence of arboreal and arbustive plants  3242 Shrubland in abandoned agricultural areas | 3210 High altitude Pastures and moorlands  3220 Shrubland  3231 Shrub and tree evolving vegetation  3232 Recent reforestation |
| Beaches dunes sand plains | 331 Beaches dunes and gravel riverbeds | 3310 Beaches dunes sand plains |
| Sparse or absent vegetation | 332 Debris accumulation and lithoid surfacing without vegetation  333 Sparse vegetation  335 Glaciers and perennial snows | 3320 Rocks, gulleys and rock surfacing  3331 Ravines  3332 Different sprse vegetation areas |
| Inland marshes | 411 Wetland and bogs vegetation | 4110 Wetlands  4120 Bogs |
| Salt marshes |  | 4211 Brackish areas  4212 Brackish valleys |
| Water courses | Riverbeds and artificial water courses | 5111 Riverbeds with sparse vegetation  5112 Riverbeds with abundant vegetation  5113 Embankments  5114 Waterways |
| Water bodies | 5121 Natural water bodies  5122 Artificial water bodies  5123 Water bodies from quarries with aquifer involvment | 5121 Natural water bodies  5122 Productive water bodies  5123 Artificial water bodies  5124 Mainland Aquaculture |
